# Genome-wide analysis of nearly all the victims of a 6200 year old massacre

**DOI:** 10.1371/journal.pone.0247332

**Published:** 2021-03-10

**Authors:** Mario Novak, Iñigo Olalde, Harald Ringbauer, Nadin Rohland, James Ahern, Jacqueline Balen, Ivor Janković, Hrvoje Potrebica, Ron Pinhasi, David Reich

**Affiliations:** 1 Centre for Applied Bioanthropology, Institute for Anthropological Research, Zagreb, Croatia; 2 Institute of Evolutionary Biology, CSIC - Universitat Pompeu Fabra, Barcelona, Spain; 3 Department of Genetics, Harvard Medical School, Boston, Massachusetts, United States of America; 4 Department of Human Evolutionary Biology, Harvard University, Cambridge, Massachusetts, United States of America; 5 Broad Institute of Harvard and MIT, Cambridge, Massachusetts, United States of America; 6 Department of Anthropology, University of Wyoming, Laramie, Wyoming, United States of America; 7 Archaeological Museum in Zagreb, Zagreb, Croatia; 8 Faculty of Humanities and Social Sciences, University of Zagreb, Zagreb, Croatia; 9 Department of Evolutionary Anthropology, University of Vienna, Vienna, Austria; 10 Howard Hughes Medical Institute, Harvard Medical School, Boston, Massachusetts, United States of America; University at Buffalo - The State University of New York, UNITED STATES

## Abstract

Paleogenomic and bioanthropological studies of ancient massacres have highlighted sites where the victims were male and plausibly died all in battle, or were executed members of the same family as might be expected from a killing intentionally directed at subsets of a community, or where the massacred individuals were plausibly members of a migrant community in conflict with previously established groups, or where there was evidence that the killing was part of a religious ritual. Here we provide evidence of killing on a massive scale in prehistory that was not directed to a specific family, based on genome-wide ancient DNA for 38 of the 41 documented victims of a 6,200 year old massacre in Potočani, Croatia and combining our results with bioanthropological data. We highlight three results: (i) the majority of individuals were unrelated and instead were a sample of what was clearly a large farming population, (ii) the ancestry of the individuals was homogenous which makes it unlikely that the massacre was linked to the arrival of new genetic ancestry, and (iii) there were approximately equal numbers of males and females. Combined with the bioanthropological evidence that the victims were of a wide range of ages, these results show that large-scale indiscriminate killing is a horror that is not just a feature of the modern and historic periods, but was also a significant process in pre-state societies.

## Introduction

Violence on a massive scale has been present in human societies for at least 13,000 years as evidenced by numerous skeletons of both sexes and all ages showing fatal violent injuries from the cemetery of Jebel Sahaba in Sudan [1–3] which is generally regarded as representing the earliest evidence of collective violence or warfare [4]. This hypothesis was additionally strengthened by the recent publication of the massacre of a group of prehistoric hunter-gatherers near Lake Turkana in Kenya [5], although some doubt the conclusion that this site represents early intragroup violence [6]. In Europe, beside the Early Neolithic Linearbandkeramik (LBK) massacre sites of Talheim [7] and Asparn/Schletz [8], several similar examples dated to prehistoric periods have been recorded [9–13]. Paleogenomic and bioanthropological studies of ancient massacres have highlighted sites where the victims were male and plausibly died all in battle [14], or were executed members of the same family as might be expected from a killing intentionally directed at subsets of a community [13], or where the massacred individuals were plausibly members of a migrant community in conflict with previously established groups [9], or where there was evidence that the killing was part of a religious ritual [15].

When dealing with such events in both ancient and modern contexts, we need a clear definition of the term “massacre”. Various definitions are used [16–20], and here we use the one proposed by Alfsdotter and colleagues [21] in their study of the massacre at Sandby borg: “an act of intentional murder upon a mass of people who were not prepared for battle, with the killing being conducted by a group”.

**Fig 1:**
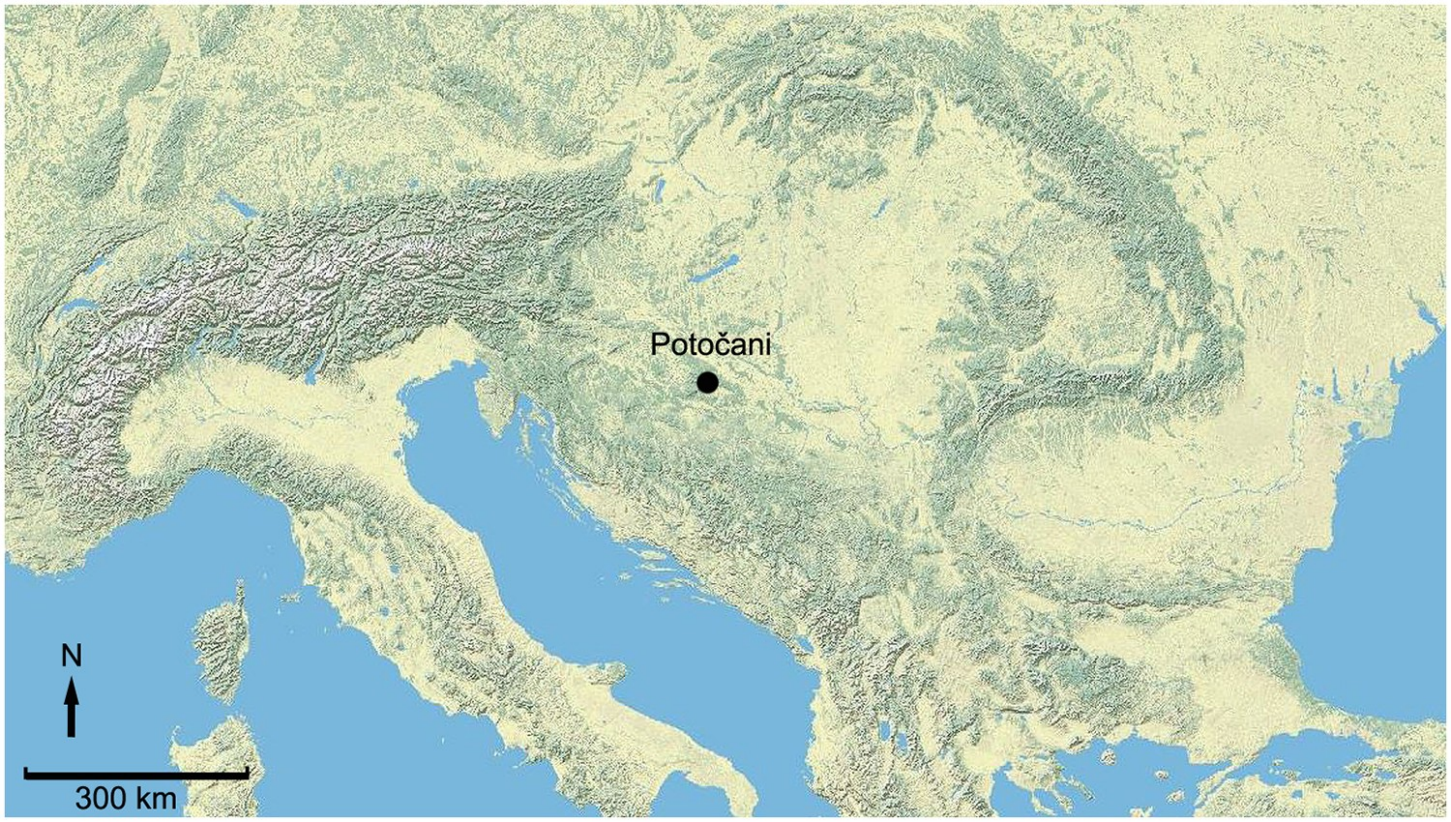
Map showing the geographic location of Potočani (base map credit: USGS National Map Viewer, http://viewer.nationalmap.gov/viewer/).

**Fig 2:**
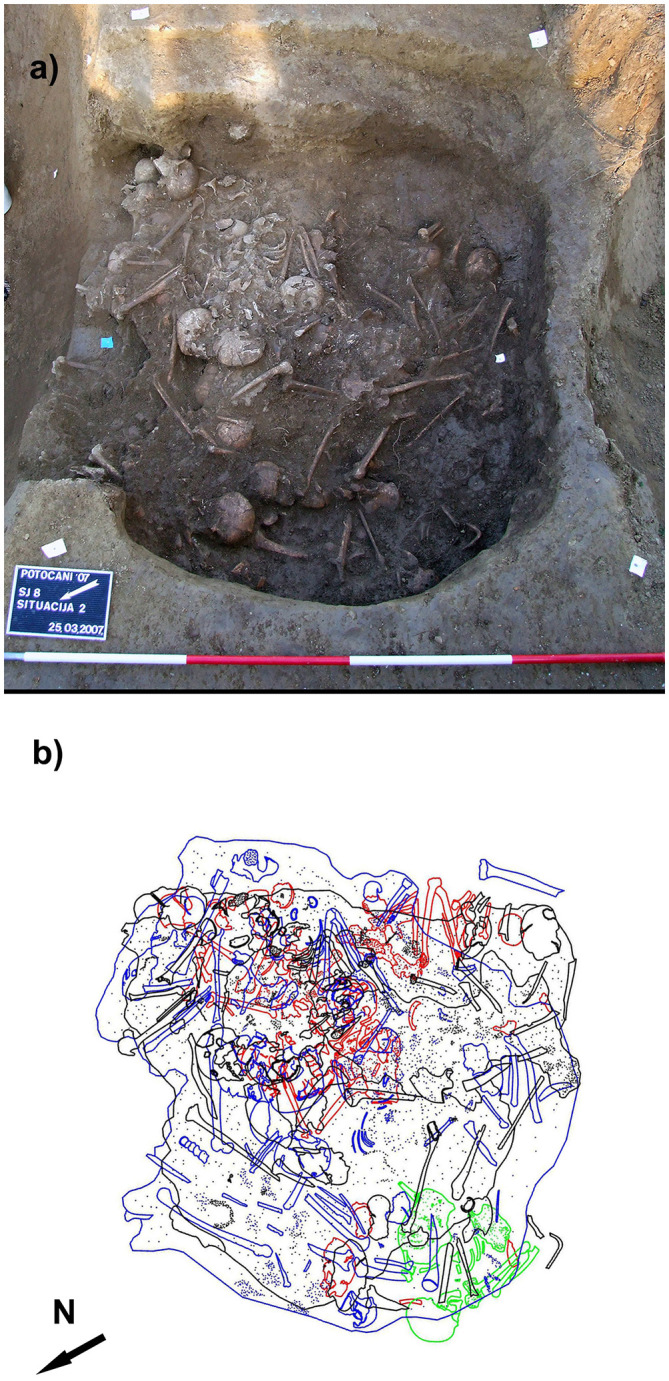
The Potočani mass burial. (A) The upper layers of the pit showing numerous commingled skeletons. (B) Schematics of the middle layer of the pit with different colors marking individual skeletons.

We provide evidence of killing on a massive scale that was not directed to a specific family by generating genome-wide ancient DNA for 38 of the 41 documented massacre victims retrieved from the Eneolithic mass burial in Potočani, continental Croatia (Fig 1). The mass burial is represented by a small pit, approx. 2 m in diameter and about 1 m in depth, containing numerous commingled, in certain cases still articulated, human skeletal remains of 41 individuals of both sexes and a wide range of ages (Fig 2a and 2b). Direct radiocarbon dates (~4200 BCE) as well as several recovered pottery fragments, assign the massacred people to the Middle Eneolithic (Copper Age) Lasinja culture which was widespread in the region of continental Croatia, northern Bosnia, Slovenia, eastern Austria, and western Hungary [22–25].

A comprehensive bioanthropological analysis of the skeletal remains from Potočani mass burial showed perimortem cranial injuries in 13 individuals (six children, three adult males and four adult females) located mostly on the side or the back of the head. The injuries caused by different weapons in combination with archaeological context and absolute dates point to a single episode of execution [22, 23].

It is generally believed that the Lasinja culture originated on the substrate of the Lengyel culture, although due to the large area it occupied as well as the additional influences, the problem of its genesis has additional complexities [26]. The Lasinja culture belongs to the Copper Age, a period when economic and social changes occurred primarily due to a series of impulses that in some way were “inspired” by the local Neolithic populations [27–30]. One of the possible reasons is the increase in the number of cattle which requires more frequent habitat changes after cattle deplete the pastures around the settlement. The importance of cattle for the Lasinja people is confirmed by zooarchaeological records suggesting that cattle husbandry played a significant, even dominant, role in peoples’ lives [31, 32]. The greater mobility probably also led to better and greater communication between different cultural groups. An important factor influencing all these events is the waning and disappearance of the Vinča culture [30]. The other important characteristic of this period is intensified copper mining and the creation of networks associated with these processes. Although there are only a few copper objects known from Lasinja sites in Croatia the people inhabiting the region during this period were familiar with copper production as they used copper objects from different deposits (carbonate and sulfide ore) [33]. Previous studies of metal objects from different Copper Age cultures and ore samples from deposits show that the circulation of excavated ore and metal followed a complex network [34].

## Results and discussion

**Fig 3:**
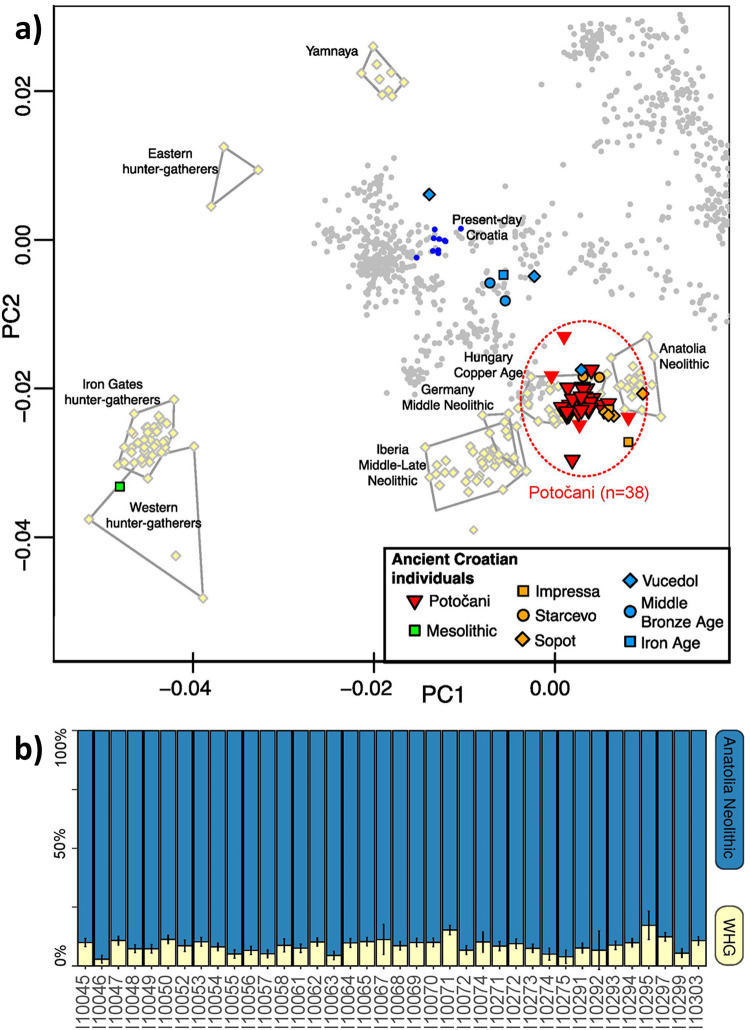
Principal Component Analysis of the Potočani individuals and mixture proportions computed with qpAdm. (A) Present-day West Eurasian individuals from the Human Origin dataset (small dots) were used to compute the principal components, with ancient individuals projected onto those coordinates. Potočani individuals with fewer than 50,000 recovered SNPs are displayed without black outline. (B) The model Anatolia Neolithic + Western hunter-gatherer was used to fit the ancestry of the Potočani individuals. The outgroup set was: Mota, Morocco Iberomaurusian, Villabruna, El Mirón, Eastern hunter-gatherers, Iran Neolithic, Levant Neolithic, Yamnaya Samara.

We generated genome-wide data from almost all individuals retrieved from the Potočani mass burial (n = 38). The genotypes have been deposited for public access to the Reich lab website (https://reich.hms.harvard.edu/datasets) and the aligned sequences to the EMBL Nucleotide Sequence Database (ENA) with the accession number PRJEB42243. Our analysis of 93% of the individuals shows that the ancestry of the people there was homogeneous. Principal Component Analysis reveals that the analyzed individuals are slightly shifted from the Anatolia Neolithic cluster in the direction of Western European hunter-gatherers, similar to other Middle to Late Neolithic European farmers before the arrival of steppe ancestry, but especially to those from Eastern Europe (Fig 3A). We confirm this pattern by successfully modelling the Potočani individuals as a mixture of predominantly Anatolian Neolithic ancestry with ~9% Western European hunter-gatherer ancestry (Fig 3B), without any evidence of steppe-related ancestry. This is further supported by the presence of paternal lineages typical of Balkan Neolithic populations (G2, I2 and C-V20), and the absence of lineages typical of steppe expansions (R1a and R1b-M269). Overall, our analysis of uniparental markers identifies 30 different mitochondrial lineages and six different Y-chromosome lineages, suggesting that the Potočani victims belonged to a large community with a diverse pool of female lineages.

**Fig 4:**
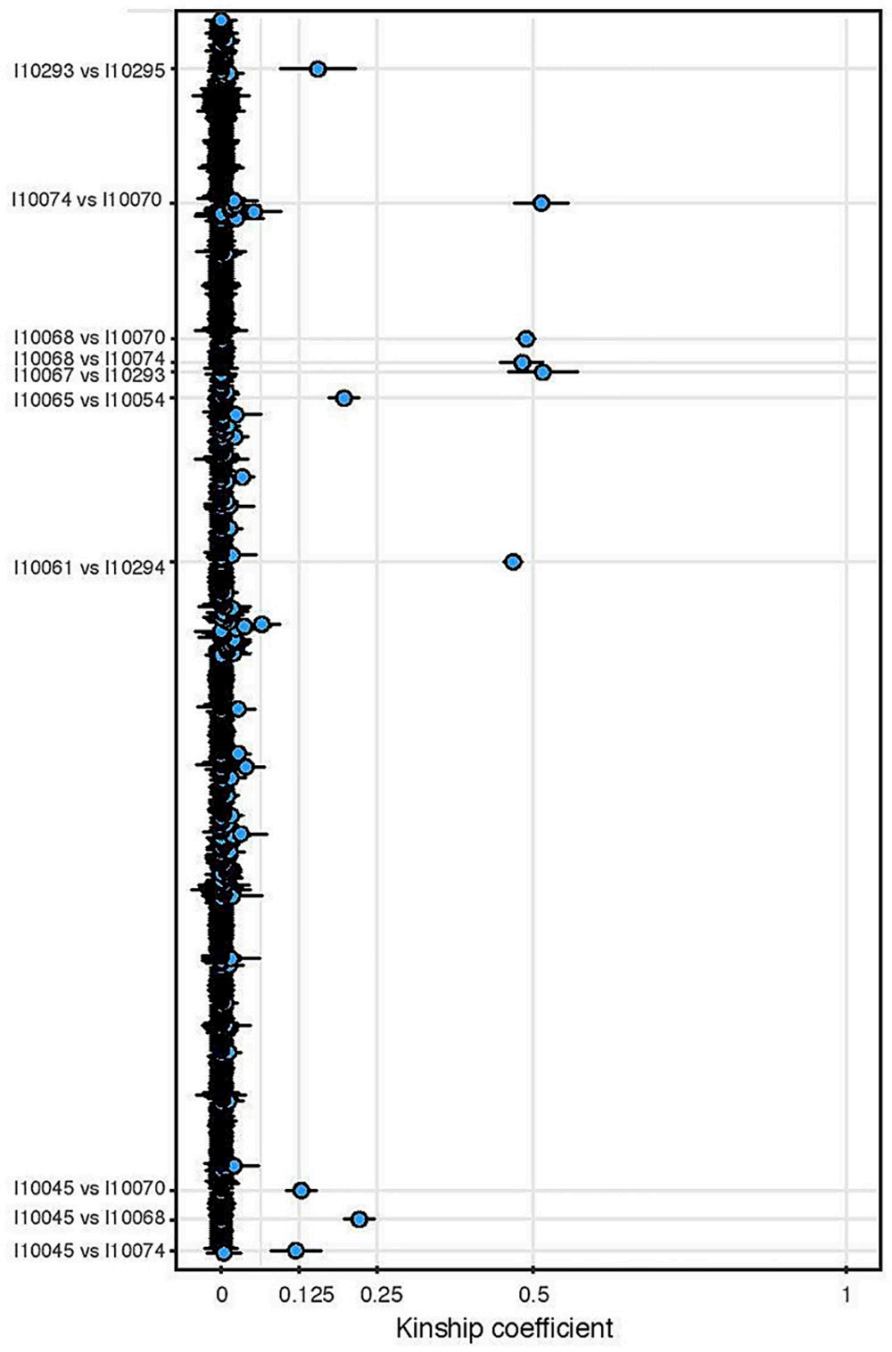
Kinship relationships among the Potočani individuals. We plot the kinship coefficient for each pairwise comparison.

**Fig 5:**
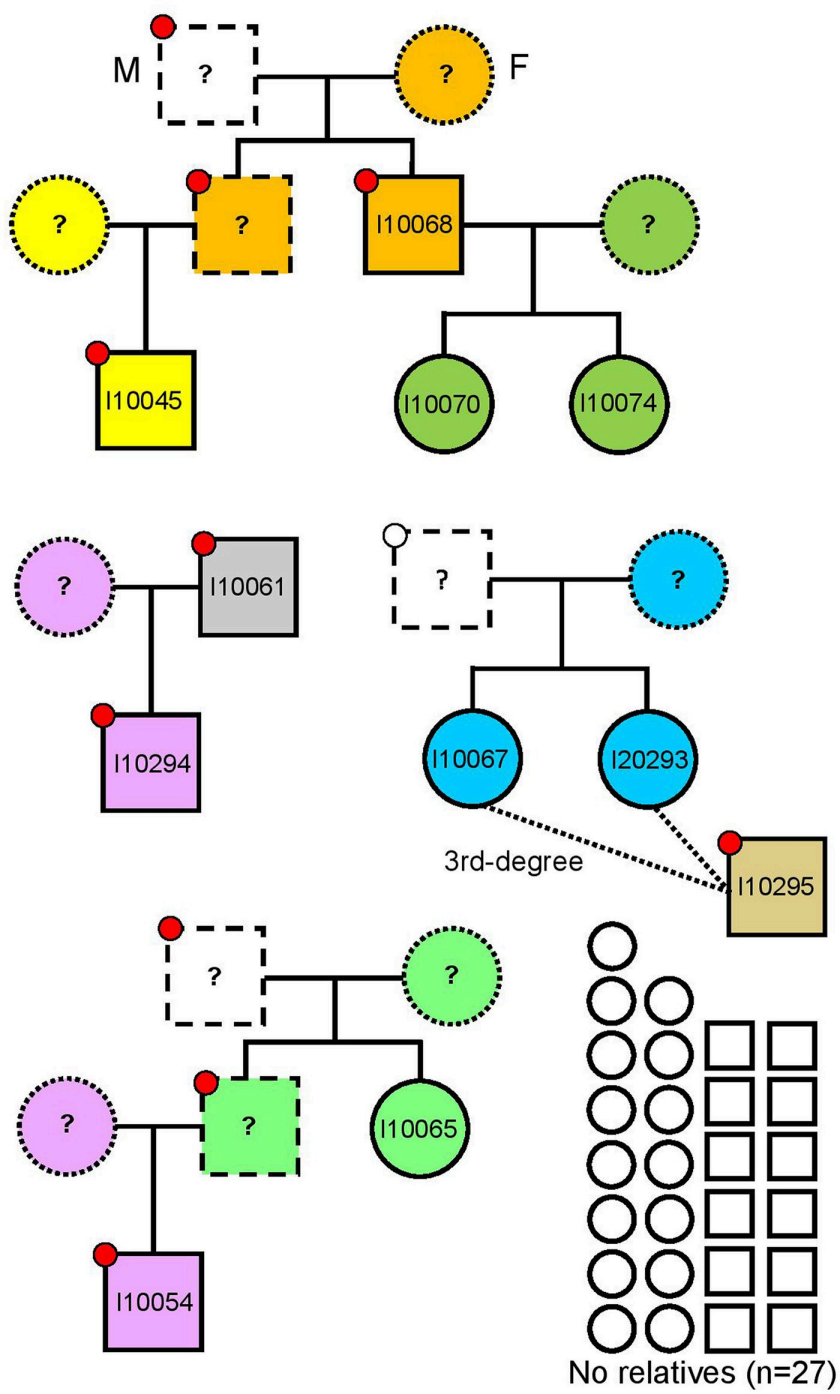
Pedigrees showing the kinship relationships among the Potočani individuals. Circles and squares represent females and males, respectively. Different mitochondrial lineages are represented by different colors inside circles and squares, and different Y-chromosome lineages are represented by different colors inside circles on the upper left corner in males. Empty circles and squares represent individuals who are not closely related (3rd-degree or closer).

Next, we studied kinship in the autosomal chromosomes and found that only 11 of the 38 analyzed individuals are closely related (3rd-degree or closer) (Fig 4), belonging to four different pedigrees (Fig 5): (i) I10068 (younger male) with his two daughters I10070 (6-10y old girl) and I10074 (11-17y old girl) and his nephew (son of his brother) I10045 (6-10y old boy), (ii) two infant sisters (I10067 and I10293, 6-10y old girls) with their 3rd-degree relative I10295 (younger male), (iii) a father I10061 (middle-aged male) and his son I10294 (11-17y old boy), and (iv) I10054 (6-10y old boy) with his paternal aunt or half-sister (same father, different mother) I10065 (younger female). Unlike the ~3000 BCE mass grave in Koszyce, Poland representing an extended family connected via several first- and second-degree relationships [13], the Potočani massacre was not targeted to a kinship group: ~70% of the analyzed individuals did not have close kin among the deceased. This suggests a violent attack targeting a small subset of individuals in a community composed of many family groups, instead of targeted killing of a small set of families within the community. The genetic analysis reveals no sex bias (20 assigned females, 18 males), showing that the massacre was also not the outcome of inter-male fighting one would expect in battles [14], and also not the result of a reprisal event targeting individuals of a specific sex.

**Fig 6:**
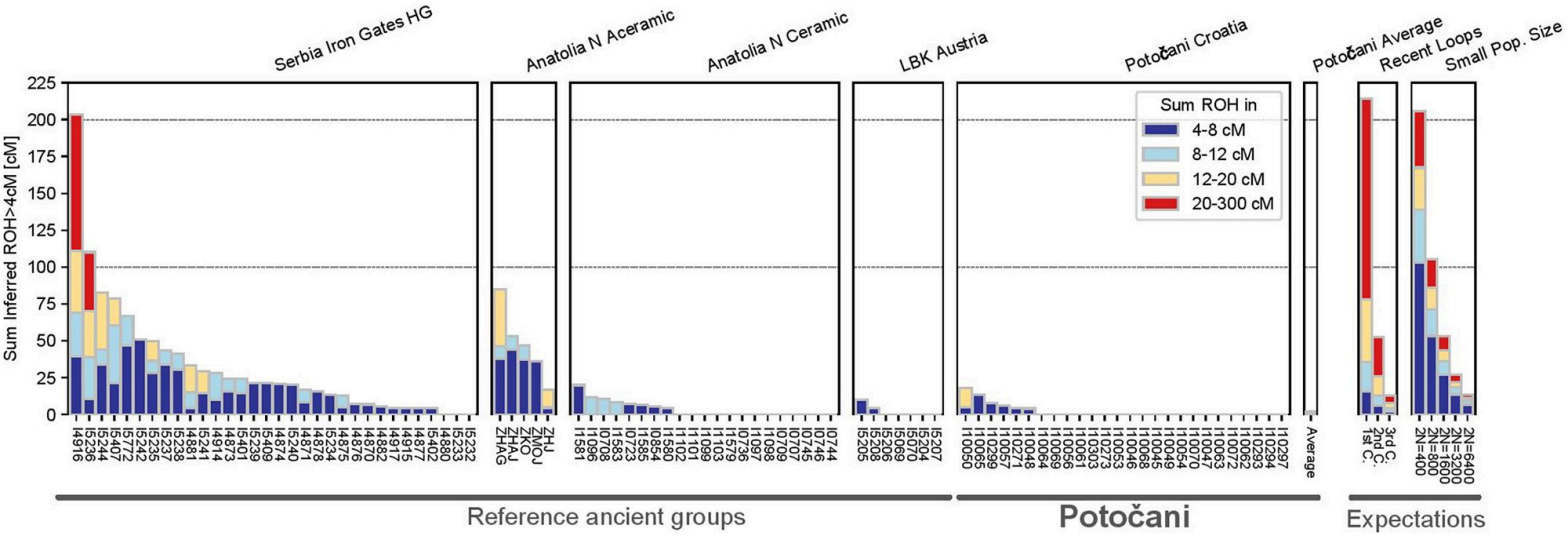
Individual ROH in Potočani individuals and reference populations. We plot ROH for each individual, summing all ROH in four length categories (stacked, colored bars). Individuals with the same population label are grouped into boxes. On the right, we depict a legend with expected ROH for (i) offspring of close kin in an outbred population (“recent loops”) and (ii) panmictic populations, using the values of Ringbauer and colleagues [35].

Analysis of runs of homozygosity reveals that the individual’s parents were in every case not closely related. Among the 27 ancient individuals with sufficient coverage for this analysis, we detected no close-kin inbreeding on the level of first or second cousins as reflected by the absence of long runs of homozygosity (>20 centimorgan). We found that 21 of the 27 individuals even had no runs of homozygosity longer than 4 cM (Fig 6); such low background relatedness points to a large local population size persisting over dozens of generations. Using the rates of runs of homozygosity 4–20 cM long and assuming a panmictic ancestry pool and a constant-sized population over the last dozens of generations, we estimate the recent effective genetic population size to 20,100–75,600 (95% CI), within the range of estimates typical for Western Eurasian populations after the transition to agriculture [35].

The mass burial from Potočani is a result of indiscriminate killing of an unrelated subset of a population with no sex and age bias, rather than a battle between two armed forces. This hypothesis is based on the demographic composition of the Potočani assemblage that includes both sexes and various age groups completely differing from the distribution seen in battle-related assemblages where younger/middle-aged males are predominant [36–41]. In terms of demographic distributions it is obvious that the Potočani assemblage is almost identical to other prehistoric massacres like Talheim [7], Asparn/Schletz [8], Schöneck-Kilianstädten [11] and Koszyce [13], but also to more recent examples [42–44] where whole or parts of communities were wiped out. The reasons for the upsurge of mass violence as well as the occurrence of massacres during the Neolithic (and Eneolithic) in Europe are “complex and multifactorial” [11]. Nevertheless, the combination of adverse climatic conditions and significant increase in population size is usually provided as the most probable reason for this phenomenon [8, 12, 45].

The examples of modern massacres show that massacres are usually processes (and not singular events) with certain patterns of violence that unfold over time and are manifested in a variety of ways [46]. In this context it is crucial to understand the events and actions leading up to and after massacres as these contribute to the emotional conditions necessary for a massacre [46]. According to some authors [e.g. 20] massacres are characterized by a mental complex which involves debasing and destroying the victims usually viewed by the perpetrators as “others”; only after this process is finished the victims are killed [20]. In other words, leaders blame a certain group for the suffering and hardships present in the society as a whole while suggesting that the situation will improve after the group is eliminated [20]. Channeling community anxiety into fear of the targeted group results in hatred for the group, the “others”, with hatred finally transforming into a desire to eliminate the feared “others”. An additional important aspect that may contribute to the massacre of innocent noncombatants is the process of identity construction and dehumanization of the “others” where they are perceived as a threat and leading to the idea that they must be destroyed in order to save society [20].

Our study is the largest-scale genetic analysis of an ancient massacre to date, and provides insight into a modality of organized violence prior to the rise of large-scale societies [8, 9, 11–13]. We find no sign of population turnover around the time of the massacre, contrasting with the pattern at massacres in the Early Neolithic Pyrenees [9] or in the Globular Amphora culture [13] where the arrival of new people likely played an important role. There is also no evidence of a sex-bias in the massacre, or age-bias or targeting of specific families, as might be expected by a reprisal and or a punitive killing. Instead, the data reveal how organized violence in this period could be indiscriminate just as indiscriminate killings have been an important feature of life in historic and modern times. An important direction for work will be to study additional massacre sites to determine the prevalence of this pattern of ancient violence.

## Materials and methods

### (Bio)archaeological context

The permissions for the archaeological fieldwork and the scientific analyses of the human skeletal remains were issued by the Conservation Department of the Ministry of Culture of the Republic of Croatia in Požega. The studied mass burial site is located in the village of Potočani in eastern Croatia; coordinates 45°38′28″N 17°23′27″E. This is the only archaeological feature found at the site as no adjoining settlement was discovered [47]. Radiocarbon dates from three human bones, taken at various horizontal positions in the pit gave near identical results of around 4200 years cal BCE (Beta-233122: 5240±40 BP; Beta-233123: 5310±40 BP; UCIAMS-140250: 5325±20 BP [25]. These dates, as well as several pottery fragments found within the pit, chronologically assign the burial to the Lasinja culture of the Middle Eneolithic (Copper Age).

The Potočani sample consists of a minimum of 41 individuals of both sexes and various age groups. In total, the Potočani population consists of 21 males and 20 females (determined genetically and confirmed morphologically). Over half of the sample (21) consisted of subadults: two younger children aged between 2 and 5 years, nine older children aged between 6 and 10 years, and ten adolescents aged between 11 and 17 years. There are 20 adults in total: 14 younger adults aged between 18 and 35 years, five middle-aged adults between 36 and 50 years old, and one adult individual whose age-at-death could not be established with certainty. The paleopathological analysis revealed low prevalence of antemortem tooth loss and carious lesions, the presence of indicators of subadult physiological stress such as cribra orbitalia (20 cases) and linear enamel hypoplasia (six cases), one case of meningitis, scurvy (five cases), and four antemortem well-healed cranial injuries recorded in three adult males and in one subadult. Numerous perimortem injuries were noted on at least 13 skulls. These exhibit a total of 28 perimortem injuries, ranging from the most frequent injury of blunt force trauma, stabbing and piercing wounds, and cuts (S1–S3 Figs). There are no visible perimortem injuries on the postcranial part of the analyzed skeletons. The number of recorded trauma per cranium varies between one and four. The distribution of observable cranial injuries does not follow a specific pattern of age and sex—the injuries were observed in one boy aged between 2 and 5 years, one girl aged between 6 and 10 years, three boys and one girl aged between 11 and 17 years, five younger adults (18–35 years; one male and four females) and two middle-aged males (36–50 years). Most of the injuries are located on the lateral, posterior, and/or superior parts of the crania [22, 23]. Nitrogen and carbon stable isotopes analysis indicates that the Potočani people are more enriched in δ^13^C and δ^15^N stable isotopes in comparison to other contemporaneous groups from continental Croatia, suggesting that they were generally consuming more animal products than their neighbors [25].

### Ancient DNA data generation

We generated ancient DNA data using a standard set of protocols [48, 49]. We isolated the cochlear portions of petrous bones and generated bone powder by sampling human petrous bones from the cranial base [50]. In dedicated clean rooms, we extracted DNA [51, 52], and generated 1–2 partially UDG-treated double-stranded libraries per sample which we distinguished from each other by using a pair of 7 base pair barcodes affixed to the ends of the ancient molecules [53]. We enriched the libraries on 96-well plates for sequences overlapping the mitochondrial genome [54] or about 1.24 million single nucleotide polymorphisms [55], and sequenced on Illumina NextSeq 500 instruments. We processed the data as previously described [48], and merged data from libraries consistent with being from the same individual. We tested for evidence of contamination based on four criteria. First we required a rate of damage in the final nucleotide of at least 3% as expected for genuine ancient DNA [53]. Second, we required a rate of match of mitochondrial sequences to the consensus sequence to have an upper bound of its 95% confidence interval of >98% [56]; three individuals did not meet this criterion (I10067, I10074, I10058) but all had low coverage in their mitochondrial DNA sequences (13-fold coverage), and had very low mismatch rates at haplotype-defining mutations covered at least twice and so we do not believe that they have evidence of mitochondrial contamination (S1 File). We also assessed the rate of polymorphism on the X chromosome (only possible in males with a minimum of 200 analyzed X chromosome positions covered at least twice) and determined that all had lower bounds for their 95% confidence intervals of <1% [57]. Fourth, we required that the ratio of Y chromosome sequences to the sum of X and Y chromosome sequences was in the range expected for this type of data for males (>35%) or females (<3%), and confirm that this was the case for all analyzed samples.

### Inferring runs of homozygosity (ROH)

We applied the software *hapROH* (downloaded from https://pypi.org/project/hapROH/, version 0.1a6) [35] to screen the ancient individuals for ROH. We used default parameters of hapROH, which are tuned to work well on 1240K SNPs and using pseudo-haploid eigenstrat file as input. As recommended, we only analyze individuals with data for at least 400,000 SNPs covered. We report the total sum of ROH >4, >8, >12, >20 centimorgan (S1 File), using the default genetic map of *hapROH*, and 5008 global haplotypes from 1000 Genomes as reference. To estimate the genetic effective population size under a panmictic model, we used the maximum likelihood method described and tested in Fernandes and colleagues [58]. Confidence intervals were obtained via the likelihood profile (1.92 units down from the maximum of the log-likelihood function provide a 95% CI).

### Inferring kinship

We looked for possible kinship relationships among the Potočani individuals using the same approach as in Olalde and colleagues [59]. In short, we computed pairwise mismatch rates by randomly sampling one allele for each individual at each 1240k SNP position. We then estimated relatedness coefficients *r*:

r=1–((x−b)/b)

with *x* being the mismatch rate for a given pair of individuals and *b* the mismatch rate expected for two unrelated individuals from the same population, divided by 2. We estimated the mismatch rate expected for two unrelated individuals by computing the median value of all the pairwise mismatch rates between Potočani individuals with more than 100,000 SNPs, assuming that most pairs are not closely related.

## Supporting information

S1 Fig Perimortem cranial trauma in two individuals from Potočani. (A) Oval-shaped blunt force trauma on the right parietal bone of individual I10050 (young adult female); the blow did not penetrate the skull. (B) Oval-shaped blunt force trauma on the right parietal bone of individual I10050 (young adult female); the blow did penetrate the skull. (C) Puncture wound on the frontal bone of individual I10052 (young adult female).
(TIF)

S2 Fig Perimortem cranial trauma in two individuals from Potočani. (A) Round-shaped blunt force trauma on the left parietal bone of individual l I10049 (11–17 years old boy); the blow did penetrate the skull. (B) Oval-shaped blunt force trauma on the occipital bone of individual I10056 (young adult female); the blow did penetrate the skull.
(TIF)

S3 Fig Perimortem cranial trauma in individual I10056 (younger female) from Potočani. Three round-shaped penetrating injuries located one above other on the right parietal and the occipital bones; the blows did penetrate the skull.
(TIF)

S1 File Extraction, library preparation and sequencing statistics of the new individuals included in the study. (XLSX)
