# Supplementary figures and images for: Genome-wide analysis of nearly all the victims of a 6200 year old massacre
Source: PLoS One. 2021 Mar 10;16(3):e0247332. doi: 10.1371/journal.pone.0247332 (PMC7946188; doi:10.1371/journal.pone.0247332)

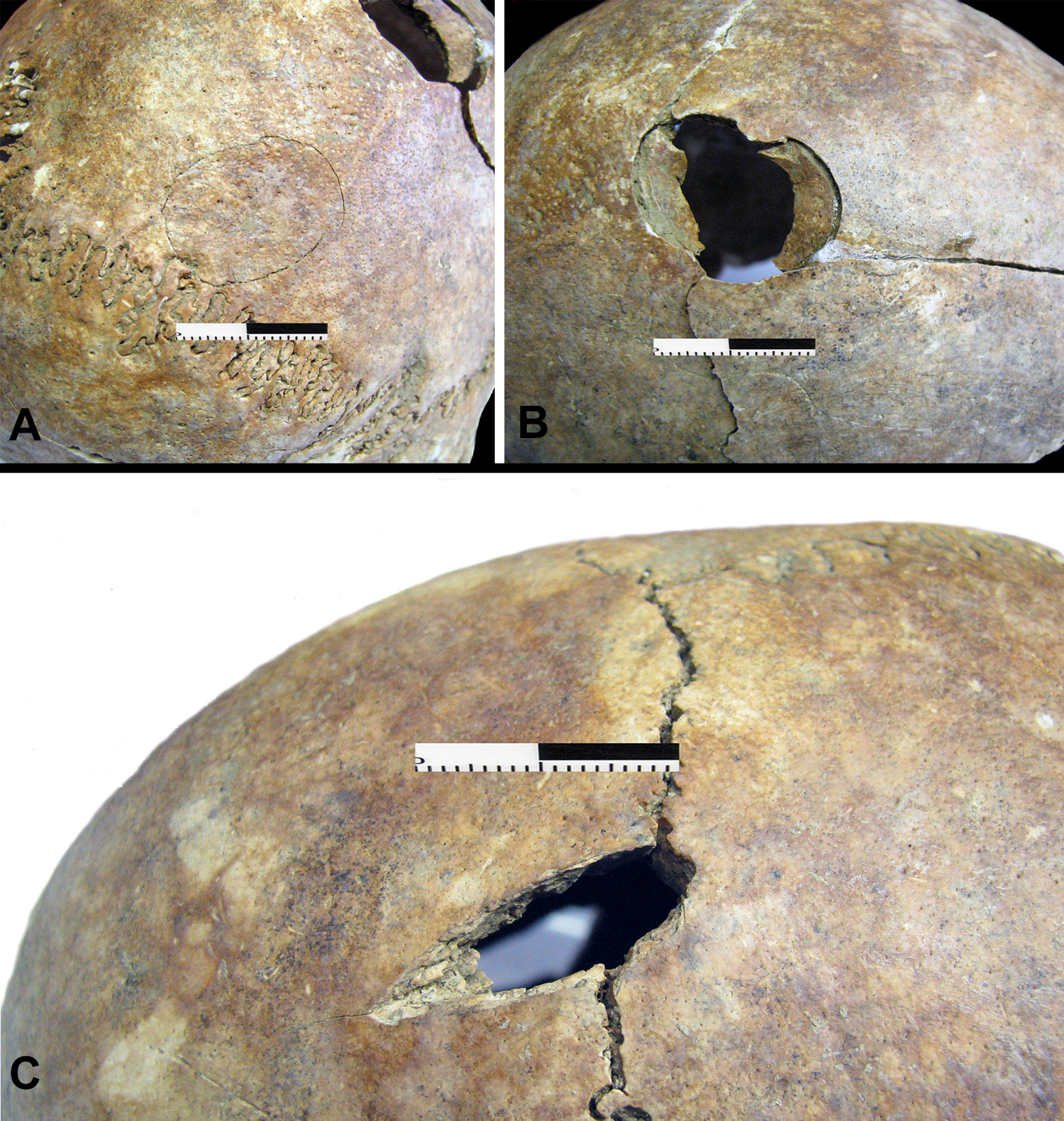

Supplement: S1 Fig — (A) Oval-shaped blunt force trauma on the right parietal bone of individual I10050 (young adult female); the blow did not penetrate the skull. (B) Oval-shaped blunt force trauma on the right parietal bone of individual I10050 (young adult female); the blow did penetrate the skull. (C) Puncture wound on the frontal bone of individual I10052 (young adult female). (TIF) [file pone.0247332.s001.tif]

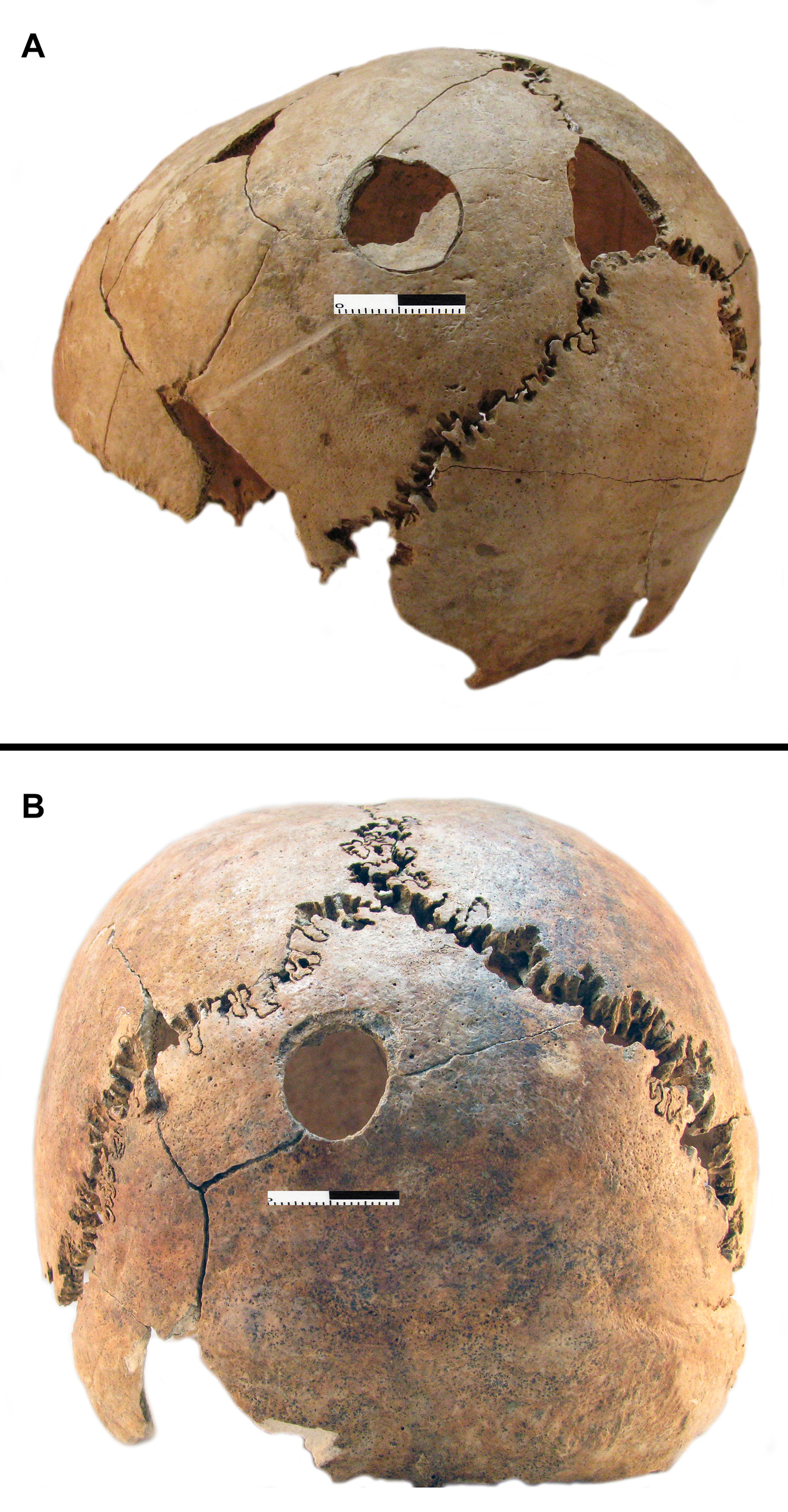

Supplement: S2 Fig — (A) Round-shaped blunt force trauma on the left parietal bone of individual l I10049 (11–17 years old boy); the blow did penetrate the skull. (B) Oval-shaped blunt force trauma on the occipital bone of individual I10056 (young adult female); the blow did penetrate the skull. (TIF) [file pone.0247332.s002.tif]

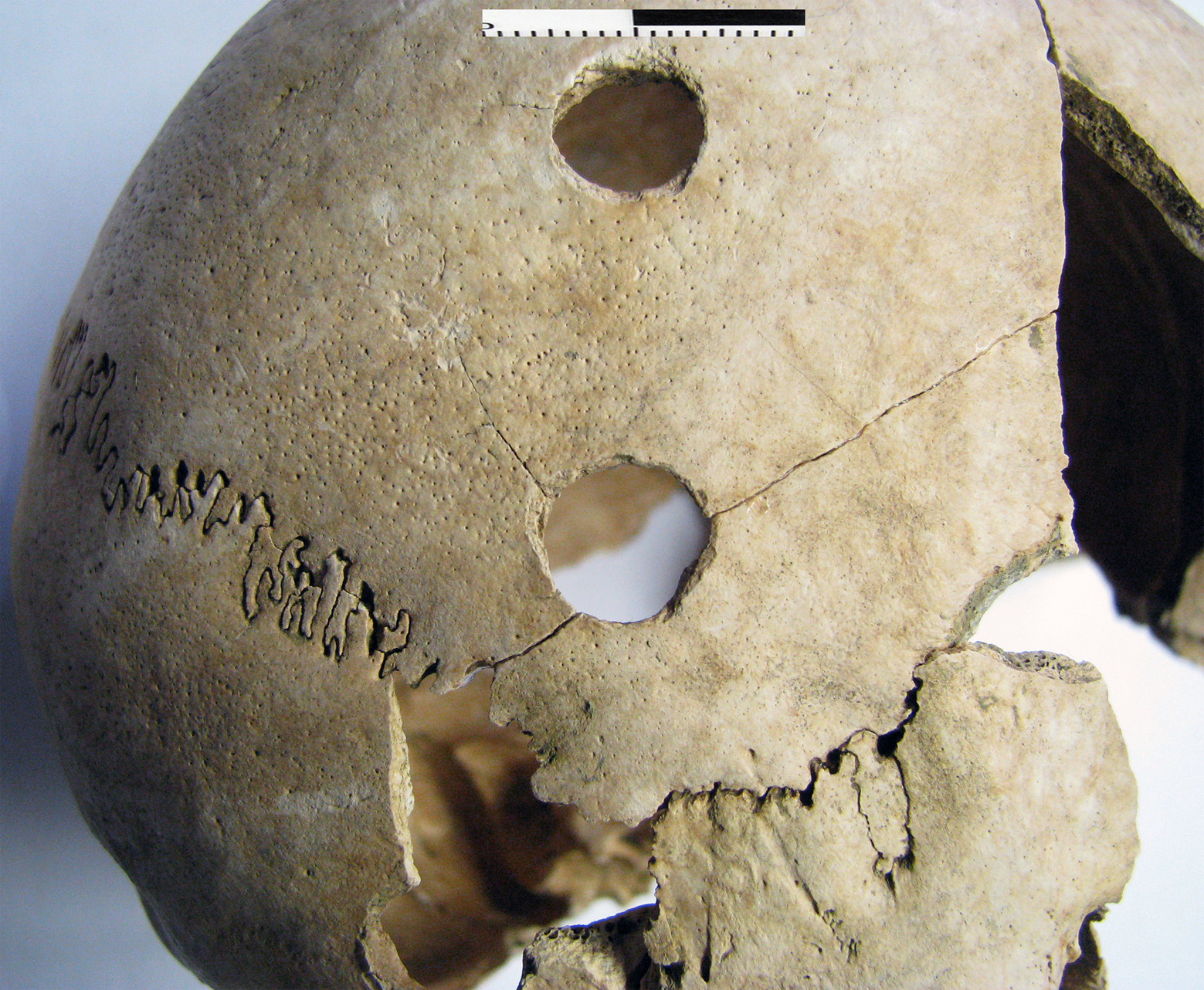

Supplement: S3 Fig — Three round-shaped penetrating injuries located one above other on the right parietal and the occipital bones; the blows did penetrate the skull. (TIF) [file pone.0247332.s003.tif]
